# SOD3 Suppresses the Expression of MMP-1 and Increases the Integrity of Extracellular Matrix in Fibroblasts

**DOI:** 10.3390/antiox11050928

**Published:** 2022-05-09

**Authors:** Jin Hyung Kim, Hae Dong Jeong, Min Ji Song, Dong Hun Lee, Jin Ho Chung, Seung-Taek Lee

**Affiliations:** 1 Department of Biochemistry, College of Life Science and Biotechnology, Yonsei University, Seoul 03722, Korea; qnt1313@yonsei.ac.kr (J.H.K.); ihaedong7@yonsei.ac.kr (H.D.J.); 2 Department of Dermatology, Seoul National University College of Medicine, Seoul 03080, Korea; minjisong@snu.ac.kr (M.J.S.); ivymed27@snu.ac.kr (D.H.L.); jhchung@snu.ac.kr (J.H.C.); 3 Laboratory of Cutaneous Aging Research, Biomedical Research Institute, Seoul National University Hospital, Seoul 03080, Korea; 4 Institute of Human-Environment Interface Biology, Seoul National University, Seoul 03080, Korea; 5 Institute on Aging, Seoul National University, Seoul 03080, Korea

**Keywords:** aging, collagen, extracellular matrix, fibroblast, MMP-1, skin, SOD3

## Abstract

The superoxide dismutase (SOD) family functions as a reactive oxygen species (ROS)-scavenging system by converting superoxide anions into hydrogen peroxide in the cytosol (SOD1), mitochondria (SOD2), and extracellular matrix (SOD3). In this study, we examined the potential roles of SOD family members in skin aging. We found that SOD3 expression levels were significantly more reduced in the skin tissues of old mice and humans than in young counterparts, but SOD1 and SOD2 expression levels remained unchanged with aging. Accordingly, we analyzed the effects of SOD3 on intracellular ROS levels and the integrity of the extracellular matrix in fibroblasts. The treatment of foreskin fibroblasts with recombinant SOD3 reduced the intracellular ROS levels and secretion of MMP-1 while increasing the secretion of type I collagen. The effects of SOD3 were greater in fibroblasts treated with the TNF-α. SOD3 treatment also decreased the mRNA levels and promoter activity of *MMP-1* while increasing the mRNA levels and promoter activities of *COL1A1* and *COL1A2*. SOD3 treatment reduced the phosphorylation of NF-κB, p38 MAPK, ERK, and JNK, which are essential for *MMP-1* transactivation. In a three-dimensional culture of fibroblasts, SOD3 decreased the amount of type I collagen fragments produced by MMP-1 and increased the amount of nascent type I procollagen. These results demonstrate that SOD3 reduces intracellular ROS levels, suppresses MMP-1 expression, and induces type I collagen expression in fibroblasts. Therefore, SOD3 may play a role in delaying or preventing skin aging.

## 1. Introduction

Skin aging can be classified into intrinsic and extrinsic aging [1,2]. Intrinsic skin aging is influenced by hormonal changes, primarily changes in estrogen levels, as well as the compactness and pigmentation levels of skin [3]. Extrinsic skin aging is affected by exogenous factors such as exposure to ultraviolet (UV) light and smoking. In both intrinsic and extrinsic skin aging, reactive oxygen species (ROS) induce several types of aging processes such as structural and functional damage to DNA, proteins, and lipids [1,2,4,5,6].

The dermis is composed of dense connective tissue that contains various components of the extracellular matrix (ECM) and is thus the most important component of skin integrity. The dermal ECM comprises fibrillar collagen, including the most abundant type I collagen, elastin, and proteoglycan [7,8]. Atrophy of the dermis is the main cause of the appearance of aged skin. The dermis contains several cell types, including fibroblasts, macrophages, and mast cells. Fibroblasts are the primary cell type in the dermis and are responsible for the production of ECM components.

Matrix metalloproteinases (MMPs) are zinc-containing endopeptidases that are involved in ECM remodeling by degrading a wide range of substrates such as collagen and other ECM proteins. The degradation of dermal ECM by MMPs contributes to wrinkling, a characteristic of skin aging [1,2]. UV irradiation and inflammation induce MMP levels via ROS generation. Elevated ROS levels result in elevated AP-1 and nuclear factor (NF)-κB activities, which in turn increase MMP expression levels in aged human skin [4,9,10]. Among MMPs, MMP-1, which is secreted by dermal fibroblasts and epidermal keratinocytes, is an important factor that accelerates skin aging by cleaving fibrillary type I and III collagens [11].

Because ROS are generally harmful to cells and tissues, the body has several defense mechanisms working against ROS. ROS scavengers consist of enzymatic and non-enzymatic systems. Enzymatic ROS scavengers include superoxide dismutase (SOD), catalase, and glutathione peroxidase [12,13,14]. Non-enzymatic ROS scavengers include ascorbic acid, vitamin A, vitamin E, α-tocopherol, and reduced glutathione [13,15]. Among these ROS scavengers, SOD has drawn attention because of its antioxidant capacity and ability to convert superoxide anions into hydrogen peroxide and oxygen, making it less harmful to cells [13,15,16].

SOD family members, including SOD1, SOD2, and SOD3, reduce free superoxide radicals in different subcellular locations [17,18,19,20]. SOD1 is mainly found in the cytoplasmic and nuclear spaces and exists in a Cu/Zn-homodimeric form. SOD2 is localized in the mitochondrial space and exists in a Mn-homotetrameric form. SOD3, also called EC-SOD, is present in the ECM and exists as a Cu/Zn-homotetramer; unlike other SODs, it scavenges extracellular superoxide anions and plays a role in maintaining redox homeostasis in tissues.

To understand the role of SOD family members in skin aging, we analyzed the expression levels of SOD members in the skin tissues of young and old mice, and the expression of SOD3 in human skin tissues. We further investigated whether SOD3 functions as a scavenger of intracellular ROS in fibroblasts. We analyzed the changes in MMP-1 and type I collagen levels induced by SOD3 in fibroblasts, as well as the signaling pathway underlying the change in MMP-1 expression caused by SOD3. Finally, under three-dimensional (3D) culture conditions of fibroblasts mimicking the dermis, we investigated whether SOD3 affects the synthesis and breakdown of type I collagen. According to these results, we demonstrated the association of SOD3 with ECM integrity and the prevention of skin aging.

## 2. Material and Methods

### 2.1. Reagents and Antibodies

Recombinant human TNF-α was purchased from Peprotech and 2′,7′-dichlorofluorescein diacetate (DCF-DA) was purchased from Sigma–Aldrich. The following antibodies were used: anti-SOD3 for western blotting (ab83108) and anti-SOD3 for immunohistochemistry (IHC; ab171738) from Abcam; anti-glyceraldehyde dehydrogenase (GAPDH; ab83108) from AbClone; anti-phospho-NF-κB inhibitor protein ɑ (IκBɑ), anti-phospho-NF-κB p65, anti-phospho-c-jun N-terminal kinase (JNK), anti-JNK, anti-phospho-p38 mitogen-activated protein kinase (MAPK) (Cell Signaling Technology), anti-NF-κB p65, anti-p38 MAPK, anti-phospho-extracellular signal-regulated kinase (ERK), and anti-ERK2 from Santa Cruz Biotechnology; and anti-collagen type I cleavage site from ImmunoGlobe. Anti-MMP-1 and procollagen α1(I) N-propeptide (pN-ColIα1) antibodies have been previously described [21]. Horseradish peroxidase (HRP)-conjugated goat anti-mouse IgG and goat anti-rabbit IgG were purchased from KOMA Biotech (Seoul, Korea). Alexa Fluor^®^ 488 goat anti-rabbit IgG (H+L) and rhodamine Red-X-conjugated goat anti-mouse IgG were purchased from Thermo Fisher Scientific (Waltham, MA, USA). SPlink HRP Detection Bulk Kit for Mouse and Rabbit Antibodies and Liquid AEC Substrate Kit (20×) for IHC were purchased from GBI labs (Bothell, WA, USA).

### 2.2. Acquisition of Mouse Skin Tissues

Dorsal skin tissues were obtained from young (3 months) and old (24 months) female albino hairless (Skh-1) mice (Orient Bio, Seongnam, Korea). All procedures involving mice were approved by the institutional animal care and use committee. The skin tissues were frozen in liquid nitrogen and stored at −70 °C for RNA and protein analysis.

### 2.3. Cell Culture

Normal human primary foreskin fibroblasts were purchased commercially from Welgene, Inc (Gyeongsan, Gyeongsangbuk, Korea). Foreskin fibroblasts were grown in Dulbecco’s modified Eagle’s medium (DMEM) (Hyclone, South Logan, UT, USA) supplemented with 10% fetal bovine serum (FBS; Gibco/Thermo Fisher Scientific, Waltham, MA, USA), and HEK293 cells were grown in DMEM supplemented with 10% bovine serum (BS; Gibco/Thermo Fisher Scientific, Waltham, MA, USA), 100 U/mL of penicillin, and 100 μg/mL of streptomycin. The cells were grown at 37 °C in 5% CO_2_ and 95% air.

### 2.4. RNA Isolation and Reverse Transcription-Polymerase Chain Reaction (RT-PCR) Analysis

Total RNA isolation, cDNA synthesis, and RT-PCR analysis were performed as previously described, with minor modifications [22]. Total RNA was isolated from mouse skin tissues and foreskin fibroblasts using TRIZOL (Invitrogen, Carlsbad, CA, USA). cDNA mixtures were synthesized from total RNA using oligo(dT)_15_ primers and AMV RTase (Promega, Madison, WI, USA), according to the manufacturer’s instructions. Amplification was performed in a final volume of 10 μL consisting of 1 pM of 5′ primer and 3′ primer of each reaction, 0.2 mM dNTPs, 1× *Taq* PCR buffer, 50 U/mL of *Taq* polymerase, and cDNAs synthesized from 0.1 µg total RNA. PCR was done for 20–35 cycles under following conditions: denaturation at 94 °C for 30 s, annealing at an annealing temperature (Table S1) for 30 s, and extension at 72 °C for 30 s. PCR products were detected by 5% polyacrylamide gel electrophoresis (PAGE) analysis and visualized by ethidium bromide staining. The expression levels of target genes were normalized to that of *GAPDH* in the corresponding sample.

### 2.5. Western Blotting Analysis

Frozen skin tissues were ground into fine powders in liquid nitrogen and homogenized after the addition of a radioimmunoprecipitation assay (RIPA) lysis buffer (50 mM Tris-HCl, pH 7.4, 150 mM NaCl, 1% NP-40, 0.5% sodium deoxycholate, 0.1% sodium dodecyl sulfate [SDS]) with a protease inhibitor cocktail (Calbiochem, San Diego, MA, USA). The homogenized skin tissues were sonicated and then centrifuged at 14,000× *g* for 20 min at 4 °C to recover proteins in the supernatant. Cells were washed twice with cold phosphate-buffered saline (PBS), lysed with the RIPA lysis buffer with 1 mM NaF, 1 mM Na_3_VO_4_, and a protease inhibitor cocktail on ice for 15 min, and centrifuged at 18,000× *g* for 15 min. SDS sample buffer (5×) was added to protein extracts from tissues, conditioned media, or cell lysates to make 1× SDS sample buffer (50 mM Tris-HCl, pH 6.8, 2% SDS, 0.1% bromophenol blue, and 10% glycerol). Protein samples were boiled in the presence of 100 mM β-mercaptoethanol and resolved using SDS-PAGE. Proteins in the gel were blotted onto polyvinylidene fluoride membrane (Millipore, Billerica, MA, USA). The blots were then incubated with the primary and secondary antibodies. Immunoreactive signals were detected with Immobilon Western HRP substrate (Millipore) and an LAS-3000 detector (Fujifilm, Tokyo, Japan).

### 2.6. IHC of Human Skin Tissues

Punch biopsy specimens (4 mm) were obtained from the buttock skin of young (woman aged 23 and men aged 24 and 28) and elderly (women aged 79 and 80 and man aged 82) individuals without current or prior skin diseases. The IHC of formalin-fixed human skin tissues was performed as described previously [23], with the exception of the anti-SOD3 antibody (1:100, ab83108). Control staining was performed with a normal goat IgG antibody that showed no immunoreactivity (data not shown). All procedures involving human subjects were approved by the institutional review board (IRB No. 1410-134-621). All participants provided written informed consent. This study was conducted in accordance with the principles of the Declaration of Helsinki.

### 2.7. Construction of hSOD3 Expression Vector

To generate pcDNA3.1-SOD3-His encoding full-length human SOD3 (GenBank NM_003102.4) with a C-terminal His tag, the *SOD3* cDNA fragment with a His tag was PCR-amplified using PrimeSTAR GXL DNA polymerase (TaKaRa, Shiga-ken, Japan) and foreskin fibroblast cDNA. The primer pair was 5′-GCG*AAGCTT*CCAGCC**ATG**CTGGCGCTACTG-3′, which includes a HindIII site (italicized) and nucleotides 90–110 of GenBank NM_003102.4 containing a start codon (bold), and 5′-GCC*GAATTC***TCA**ATGATGATGATGATGATGGGCGGCCTTGCACTCGCTC-3′, which includes an EcoRI site (italicized), a stop codon (bold), His tag (underlined), and nucleotides 815-797 of GenBank NM_003102.4. The PCR product was cleaved with HindIII and EcoRI and ligated into the HindIII and EcoRI sites of the pcDNA3.1 vector.

### 2.8. Purification of Human SOD3-His from HEK293 Cells

HEK293 cells were transfected with pcDNA3.1-hSOD3-His using the calcium phosphate method, followed by selection with 1200 μg/mL of G418 to generate HEK293 cells that stably expressed human SOD3-His (HEK293-SOD3-His). Subconfluent HEK293-SOD3-His cells were incubated in a serum-free medium for 3.5 d. Proteins in the conditioned medium were precipitated using 80% ammonium sulfate. The pellet was resuspended in Tris-buffered saline (TBS) (20 mM Tris-HCl, pH 7.4, 150 mM NaCl). Human SOD3-His protein was purified from the resuspended sample using Ni^2+^-NTA agarose (QIAGEN, Hilden, Germany) and dialyzed with TBS, as described previously [24].

### 2.9. Detection of Cellular ROS Levels

Foreskin fibroblasts treated with TNF-α and/or SOD3 were washed twice with PBS and treated with 10 μM of DCF-DA in a serum-free DMEM in a dark state at 37 °C in 5% CO_2_ incubator for 30 min. The cells were then washed twice with PBS, once with phenol red-free DMEM (Sigma-Aldrich, St. Louis, MO, USA), and analyzed via fluorescence microscopy. After DCF-DA assay, cells were fixed with 3.7% paraformaldehyde in PBS and stained with 0.005% crystal violet. The stained cells were lysed with 1% SDS, and the absorbance was measured at 600 nm [25]. The intensity of fluorescence in micrograms was measured using the ImageJ software (National Institutes of Health, Bethesda, MD, USA).

### 2.10. Reporter Constructs for Human COL1A1 and COL1A2 Promoters and MMP-1 Promoter

The luciferase reporter construct of the human *COL1A1* promoter, pGL3-COL1A1 promoter, harbors a 2378-bp 5-flanking sequence of the human *COL1A1* gene (−2322 to +41; +1 from the upstream of the transcription start site), which was PCR-amplified using PrimeSTAR GXL DNA polymerase, and primer pair: 5′-AT*GGTACC*TGTCACACGCCAGGCACACA-3′, which includes a KpnI site (italicized) and nucleotides 50203941-50203960 of GenBank NC_000017.11, and 5′-GAC*GCTAGC*TGCTCCGACCCCGAGGAGA-3′, which includes an NheI site (italicized) and nucleotides 50201600-50201618 of GenBank NC_000017.11. The luciferase reporter construct of the human *COL1A2* promoter, pGL3-COL1A2 promoter included a 3577-bp 5′-flanking sequence of the human *COL1A2* gene (−3539 to +38; +1 from the upstream of the transcription start site), which was PCR-amplified using PrimeSTAR GXL DNA polymerase and primer pair: 5′-AT*GGTACC*GCCTGGGTGATAAAGTGAGACCTTCTC-3′, which includes a KpnI site (italicized) and nucleotides 94391305-94391331 of GenBank NC_000007.14, and 5′-GAC*GCTAGC*GGCAAAAGTTTCCCTGGCC-3′, which includes an NheI site (italicized) and nucleotides 94394842-94394860 of GenBank NC_000007.14. The PCR products were cleaved with KpnI and NheI, and then ligated into the KpnI and NheI sites of the pGL3-Basic plasmid. A luciferase reporter construct of the human *MMP-1* promoter, pGL3-MMP-1 promoter, containing a 1938-bp 5′-flanking sequence of the human *MMP-1* gene (−1880 to +40; GenBank Accession No. NM_002421.4) has been described previously [9].

### 2.11. Dual-Luciferase Reporter Assay

Transfection of reporter genes into foreskin fibroblasts was performed using Lipofectamine LTX (Thermo Fisher Scientific) as described previously, with minor modifications [9]. Foreskin fibroblasts (5 × 10^4^ cells/well) were seeded in 24-well plates, and the medium was replaced with fresh DMEM supplemented with 10% FBS. Promoterless pGL3-Basic (0.5 μg) or pGL3-MMP-1, COL1A1, or COL1A2 promoter (0.5 μg each) encoding firefly luciferase driven by the *MMP-1*, *COL1A1*, or *COL1A2* promoter, respectively, and pRL-TK (0.05 μg, Promega) encoding Renilla luciferase driven by the herpes simplex virus thymidine kinase promoter in 25 µL Opti-MEM were incubated with Lipofectamine LTX (0.75 µL, Invitrogen) and PLUS reagent (0.25 µL, Invitrogen) in 25 µL Opti-MEM (Gibco/Thermo Fisher Scientific) for 20 min at room temperature. Cells were treated with this mixture for 5 h and then incubated with DMEM supplemented with 10% FBS for 24 h. The cells were then treated with SOD3 in a serum-free medium for 24 h. Luciferase activity was measured using the dual-luciferase reporter assay system (Promega), and firefly luciferase activity in transfected cells was normalized to Renilla luciferase activity.

### 2.12. Analysis of Collagenolysis and Collagen Synthesis in a 3D Culture System

Collagenolysis and collagen synthesis in a 3D culture system were analyzed as described previously, with minor modifications [9,23]. Foreskin fibroblasts (8 × 10^5^ cells/mL) were trypsinized and resuspended in rat tail collagen I solution (2.8 mg/mL; Corning Inc., Corning, NY, USA): 5× DMEM:10× reconstitution buffer (260 mM NaHCO_3_, 200 mM HEPES, and 50 mM NaOH) at a ratio of 7:2:1, and then 0.15 mL of the cell mixture was placed in a glass-bottom (35 mm × 10 mm, hole 13 φ) dish (SPL Life Sciences, Pocheon, Korea). After gel polymerization for 1 h at 37°C, 1.5 mL of phenol red-free DMEM with or without TNF-α and/or SOD3 was added, and collagen-embedded cells were incubated for 24 h at 37 °C in an atmosphere of 5% CO_2_ and 95% air. The cells were fixed with 3.7% paraformaldehyde for 30 min and permeabilized in 0.2% Triton-X 100 for 10 min. Cells were incubated with Hoechst 33258 (2 μg/mL) for 30 min for nuclear staining, blocked in 3% bovine serum albumin for 30 min, and immunostained overnight at 4 °C with rabbit anti-type I collagen cleavage-site antibody (2.5 μg/mL) to detect collagenolysis and mouse anti-pN COL1A1 antibody (1:20) to detect type I collagen synthesis. The cells were then washed with PBS and incubated for 2 h with Alexa Fluor^®^ 488 goat anti-rabbit IgG (H+L) (Invitrogen) and anti-mouse IgG (H+L) Rhodamine Red-X (1 U/mL). Images were obtained using a confocal microscope (LSM880; Carl Zeiss, Oberkochen, Germany) with a 10× Plan-Apochromat objective lens and the Zen software (Carl Zeiss). The excitation wavelengths were 405 nm for Hoechst 33258, 488 nm for Alexa Fluor^®^ 488, and 555 nm for rhodamine Red-X. To avoid bias during image acquisition, all images were obtained from randomly selected fields using the same parameters, including exposure time, laser power, and offset settings. The fluorescence intensity was determined using ImageJ software.

### 2.13. Statistical Analyses

All data are presented as the mean ± standard deviation of at least three independent experiments. Statistical significance was analyzed using an unpaired two-tailed Student’s *t*-test. A *p*-value < 0.05 was considered to indicate statistical significance.

## 3. Results

### 3.1. SOD3 Levels Decrease during Skin Aging

**Figure 1:**
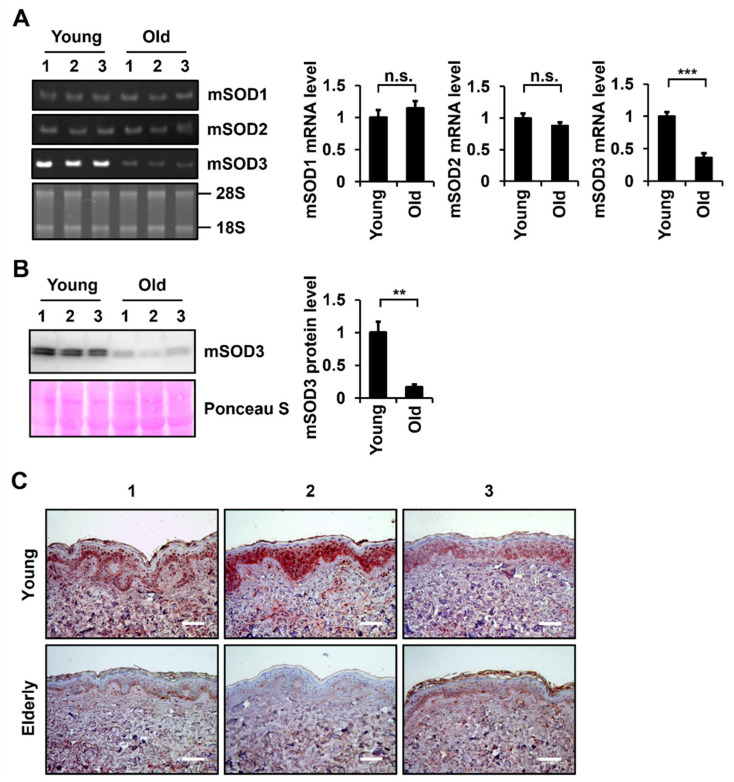
Determination of the expression levels of SOD family members in young and old skin tissues of mice and humans. (**A**) mRNA levels of *SOD1*, *SOD2*, and *SOD3* in mouse skin tissues were determined using RT-PCR. The amount of RNA was normalized by 18S and 28S RNA. (**B**) SOD3 protein levels in mouse skin tissues were determined via western blotting using an antibody against SOD3. The amount of protein was normalized by total protein amount using Ponceau S staining. Graphs show Sod mRNA and protein levels relative to the levels in young tissues, which were quantified using the ImageJ software. Values are presented as the means ± standard deviations (*n* = 3). ** *p* < 0.01 and *** *p* < 0.001 vs. young tissues. n.s.: not significant. (**C**) IHC analysis of SOD3 protein levels in young and elderly human skin tissues (*n* = 3 each). Sections of skin tissues were stained with SOD3 antibody and horseradish peroxidase-conjugated secondary antibody with 3-amino-9-ethylcarbazole, and counterstained with hematoxylin. Magnification, ×200. Bar = 100 µm.

To understand the role of SOD family members in skin aging, mRNA levels of *Sod* members in the skin tissues of young hairless mice (3 months old) and old hairless mice (24 months old) were determined via RT-PCR analysis. *Sod1* and *Sod2* mRNA levels were not significantly altered in the skin tissues of young and old mice, but a significant decrease in *Sod3* mRNA levels was detected in the skin tissues of old mice compared to those of young mice (Figure 1A). To detect differences in Sod3 protein levels in the skin tissues of young and old mice, proteins were extracted from skin tissues and analyzed via western blotting. Similar to the RT-PCR results, the protein expression levels of SOD3 were significantly reduced in old mouse skin tissues compared to young mouse skin tissues (Figure 1B).

To examine the age-dependent changes in SOD3 levels in human skin, IHC analysis was performed on UV-protected buttock skin tissues from young (aged 25.0 ± 2.6) and elderly (aged 80.3 ± 1.5) individuals. SOD3-positive signals were present in both the epidermis and dermis of young skin tissues (Figure 1C). SOD3 staining was intense in the basal and spinous layers of the epidermis and in fibroblasts and endothelial cells of the dermal region. The elderly skin tissues also showed a similar pattern of SOD3 staining as the young skin tissues. However, less SOD3-positive cells and reduced SOD3 signals were observed in the epidermis and dermis of elderly skin tissues than those of the young skin tissues (Figure 1C).

### 3.2. SOD3 Treatment Attenuates ROS Generation in Fibroblasts

**Figure 2:**
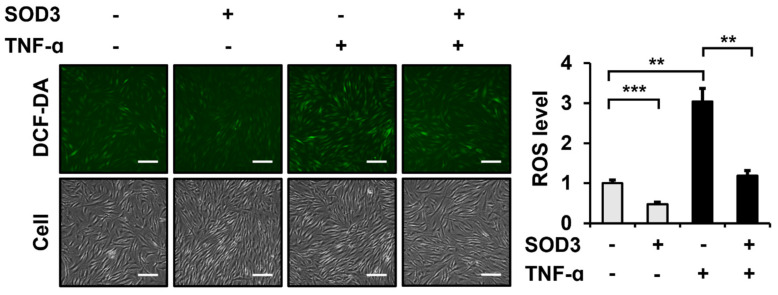
Effect of recombinant SOD3 treatment on the intracellular ROS levels in fibroblasts. Human foreskin fibroblasts were incubated in a serum-free medium in the presence of TNF-α (1 ng/mL) and/or SOD3 (3 μg/mL) for 24 h. Intracellular ROS levels were measured by incubating the serum-free medium with 10 μM DCF-DA for 30 min and detected as green fluorescence at 525 nm. ROS levels were normalized using crystal violet-stained cell counts. The graph shows ROS levels relative to the levels in untreated cells, which was assessed using the ImageJ software. Each value represents the mean ± standard deviation of three independent experiments. ** *p* < 0.01 and *** *p* < 0.001 vs. control. Magnification, ×200. Bar = 100 µm.

SOD3 acts as a ROS scavenger in the extracellular space [18]. Therefore, we analyzed whether SOD3 affected intracellular ROS levels in foreskin fibroblasts. As a source of SOD3, recombinant human SOD3 with a C-terminal His_6_ tag (SOD3-His) was expressed in HEK293 cells and purified (Figure S1). Intracellular ROS levels were measured via DCF-DA analysis. The incubation of fibroblasts with SOD3-His (3 µg/mL) for 24 h significantly reduced ROS production to 47.4 ± 6.0% (Figure 2). The treatment of fibroblasts with TNF-α (1 ng/mL) increased the ROS levels to 304.0 ± 33.1%. The ROS scavenging effect of SOD3 (39.2 ± 4.1% for SOD3 (+) relative to SOD3 (−)) in TNF-α-treated cells was comparable to that (47.4 ± 6.0% for SOD3 (+) relative to SOD3 (−)) in cells not treated with TNF-α.

### 3.3. SOD3 Treatment Decreases MMP-1 Secretion and Increases Type I Collagen Secretion in Fibroblasts

**Figure 3:**
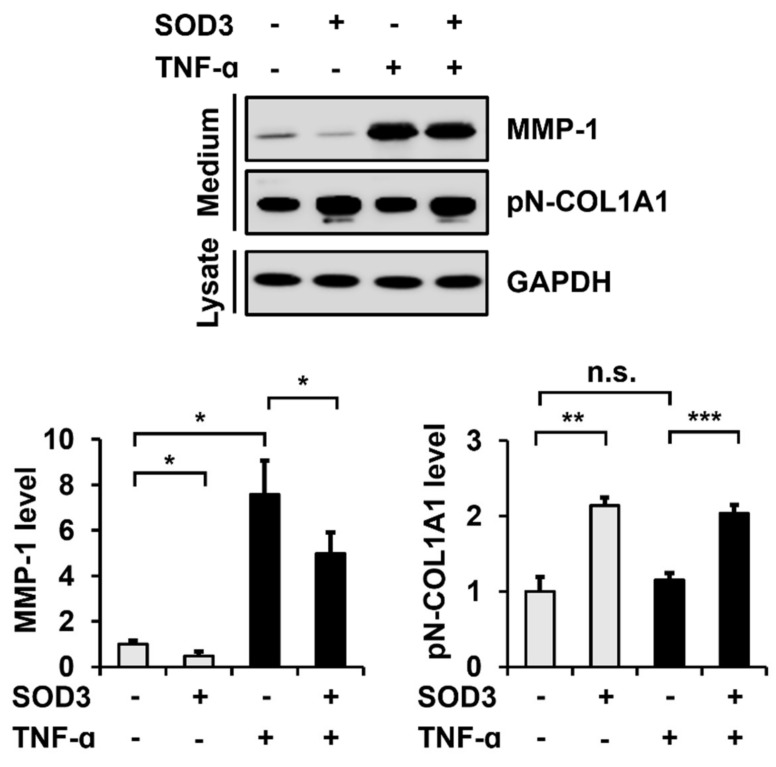
Effect of SOD3 on the secretion of matrix metalloproteinase (MMP)-1 and type I collagen in fibroblasts. Human foreskin fibroblasts were incubated in a serum-free medium in the presence of TNF-α (1 ng/mL) and/or SOD3 (3 μg/mL) for 24 h. The secreted levels of MMP-1 and type I collagen in the conditioned media were assessed via western blot analysis using antibodies against MMP-1 and pN-COL1A1, respectively. GAPDH levels in cell lysates were analyzed as a loading control. Graphs show the levels of MMP-1 and type I collagen relative to the levels in non-treated cells, which were quantified using the ImageJ software. Each value represents the mean ± standard deviation of three independent experiments. * *p* < 0.05, ** *p* < 0.01, and *** *p* < 0.001 vs. control. n.s.: not significant.

We then examined the effects of SOD3 on MMP-1 and type I collagen secretion in foreskin fibroblasts. Incubation of SOD3-His in fibroblasts significantly decreased the secretion of MMP-1 (48.1 ± 18.0%) and increased the secretion of type I collagen (213.7 ± 11.0%) (Figure 3). The presence of TNF-α (1 ng/mL) enhanced the MMP-1 secretion to 756.8 ± 150.9%, but did not significantly alter the type I collagen secretion. The decrease in the MMP-1 secretion by SOD3 (69.2 ± 15.2%) in TNF-α-treated cells was also significant, comparable to that (48.1 ± 18.0%) in non-TNF-α-treated cells.

### 3.4. SOD3 Treatment Downregulates MMP-1 Expression and Upregulates Type I Collagen Expression at the Transcriptional Level

**Figure 4:**
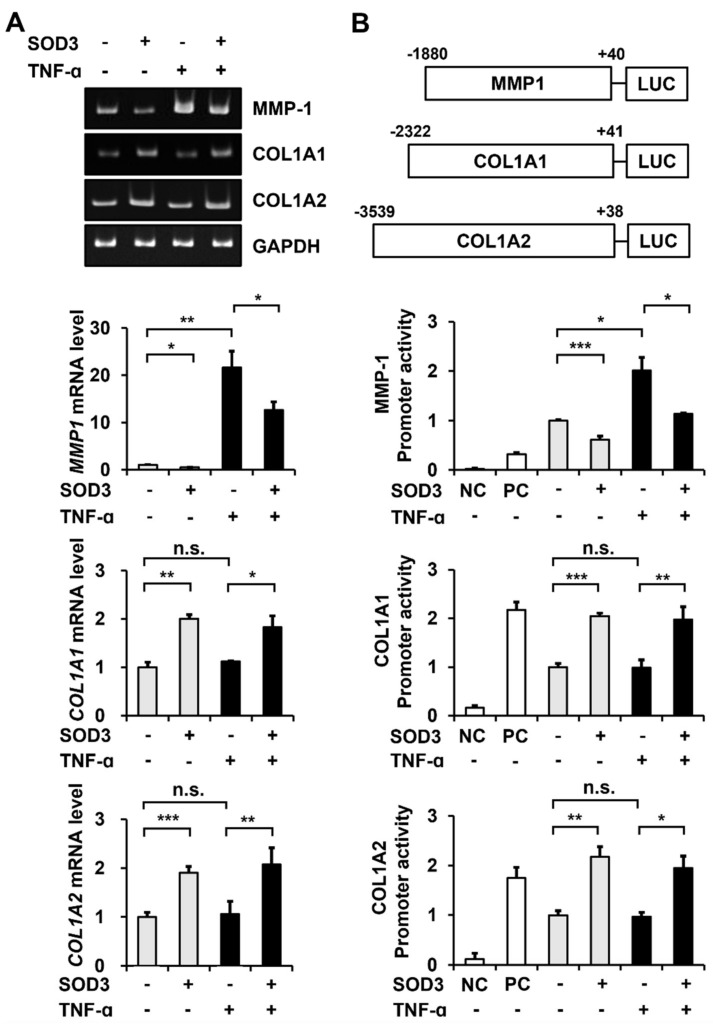
Effect of SOD3 on the transcription of *MMP-1* and type I collagen genes in fibroblasts. (**A**) Human foreskin fibroblasts were incubated in a serum-free medium in the presence of TNF-α (1 ng/mL) and/or SOD3 (3 μg/mL) for 24 h. Levels of *MMP-1*, *COL1A1*, and *COL1A2* mRNAs were evaluated via conventional and real-time RT-PCR analysis. Graphs show *MMP-1, COL1A1*, and *COL1A2* mRNA levels relative to the levels in non-treated cells determined via the real-time RT-PCR analysis. (**B**) Dual-luciferase reporter assay was performed to measure the *MMP-1*, *COL1A1*, and *COL1A2* promoter activities in the presence of TNF-α and/or SOD3. Schematic representation of luciferase reporter constructs driven by *MMP-1*, *COL1A1*, and *COL1A2* promoters is shown. The numbers of *MMP-1*, *COL1A1*, and *COL1A2* promoters indicate the nucleotide positions labeling the transcription site as +1 and its upstream as −1. Foreskin fibroblast cells were transfected with pRL-TK and promoter-less pGL3-Basic (negative control: NC), pGL3-Promoter containing SV40 promoter (positive control: PC), or pGL3-MMP-1 promoter and COL1A1 or COL1A2 promoter. Luciferase activity was expressed as a relative ratio of firefly luciferase activity measured at 560 nm to Renilla luciferase activity measured at 480 nm. Graphs show relative luciferase levels by *MMP-1*, *COL1A1*, and *COL1A2* promoters relative to the levels in non-treated cells. Each value is the mean ± standard deviations of three independent experiments. * *p* < 0.05, ** *p* < 0.01, and *** *p* < 0.001 vs. control. n.s.: not significant.

To determine whether the observed SOD3-mediated decrease in MMP-1 secretion and increase in type I collagen secretion were due to the suppression of *MMP-1* transcription and induction of *COL1A1* and *COL1A2* transcription, we performed RT-PCR and reporter gene assays in foreskin fibroblasts treated with or without SOD3-His. Conventional and real-time RT-PCR analyses showed that the *MMP-1* mRNA levels decreased to 53.3 ± 4.4% and *COL1A1* and *COL1A2* mRNA levels increased to 200.4 ± 8.5% and 190.3 ± 13.0%, respectively, in SOD3-treated cells (Figure 4A). Treatment with TNF-α increased the *MMP-1* mRNA level to 2165.4 ± 343.0% but did not significantly change *COL1A1* and *COL1A2* mRNA levels. Consistent with the results for MMP-1 and type I collagen secretion, SOD3 decreased the *MMP-1* mRNA level to 58.3 ± 8.1% and increased *COL1A1* and *COL1A2* mRNA levels to 162.6 ± 21.4% and 196.4 ± 32.2%, respectively, in TNF-α-treated cells.

Treatment with SOD3 reduced the *MMP-1* promoter-driven luciferase activity to 61.2 ± 7.2% and increased *COL1A1* and *COL1A2* promoter-driven luciferase activities to 204.7 ± 5.6% and 217.4 ± 20.3%, respectively (Figure 4B). TNF-α stimulation increased the *MMP-1* promoter activity to 201.5 ± 26.1%, but did not significantly change *COL1A1* and *COL1A2* promoter activities. In TNF-α-stimulated cells, treatment with SOD3 decreased the *MMP-1* promoter activity to 56.5 ± 0.6% and increased *COL1A1* and *COL1A2* promoter activities to 199.6 ± 27.1% and 200.9 ± 24.8%, respectively. These data demonstrate that SOD3 suppresses the expression of MMP-1 and induces the expression of type I collagen at the transcriptional level.

### 3.5. SOD3 Treatment Downregulates MMP-1 Expression by Inhibiting AP-1 and NF-κB Signaling Pathways in Fibroblasts

**Figure 5:**
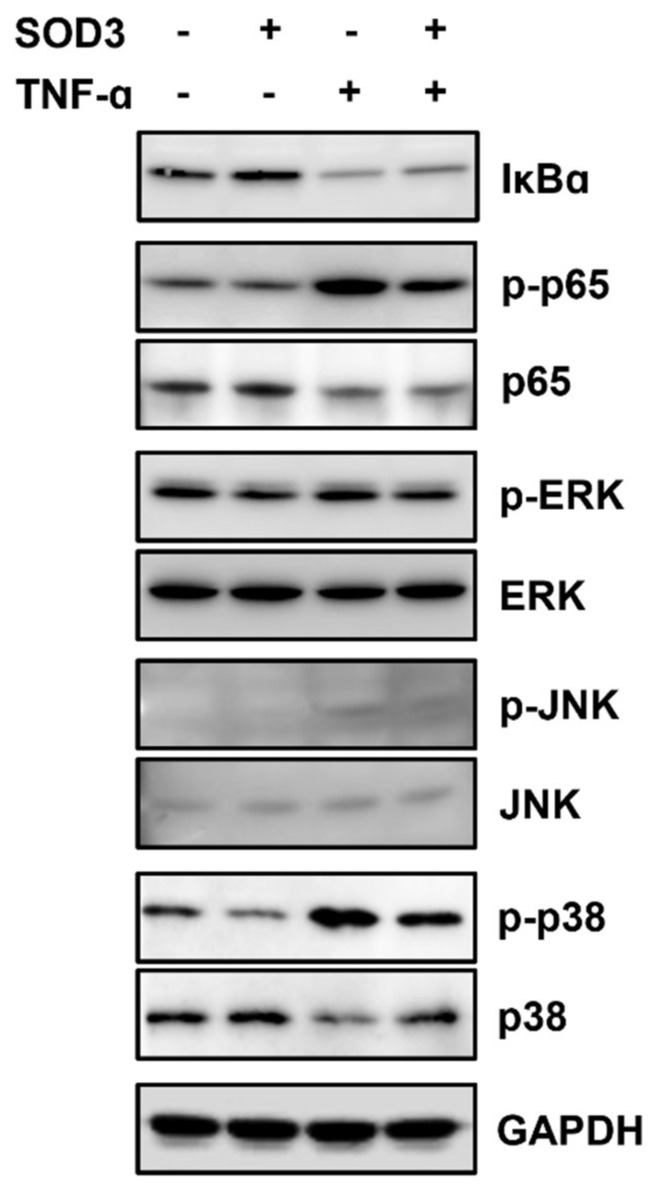
Effects of SOD3 on the level of IκB and phosphorylation of NF-κB p65 and MAPKs in fibroblasts. Human foreskin fibroblasts were incubated without serum in the presence of TNF-α (1 ng/mL) and/or SOD3 (3 μg/mL) for 12 h. The phosphorylation and expression levels of IκBα, NF-κB p65, ERK, JNK, and p38 MAPK were assessed via western blotting analysis.

SOD3 treatment significantly reduced MMP-1 expression both in the absence and presence of TNF-α. *MMP-1* transcription is upregulated by the activation of ERK, JNK, p38 MAPK, and NF-κB signaling pathways [26,27]. Therefore, to investigate how SOD3 suppresses *MMP-1* transactivation, changes in these signaling proteins were analyzed in foreskin fibroblasts incubated with or without SOD3-His and TNF-α. Treatment with SOD3 increased IκB levels and decreased phosphorylation of the p65 subunit of NF-κB, ERK, and p38 MAPK (Figure 5). The presence of TNF-α decreased IκB levels and increased the phosphorylation of NF-κB p65, JNK, and p38 MAPK. In TNF-α-treated cells, SOD3 reversed the TNF-α-induced signaling pathways. These results indicate that SOD3 downregulates MMP-1 expression by inhibiting the transcription factors AP-1 and NF-κB, which play important roles in *MMP-1* transactivation.

### 3.6. SOD3 Treatment Reduces the Breakdown and Increases the Production of Type I Collagen in 3D Cultures of Fibroblasts

**Figure 6:**
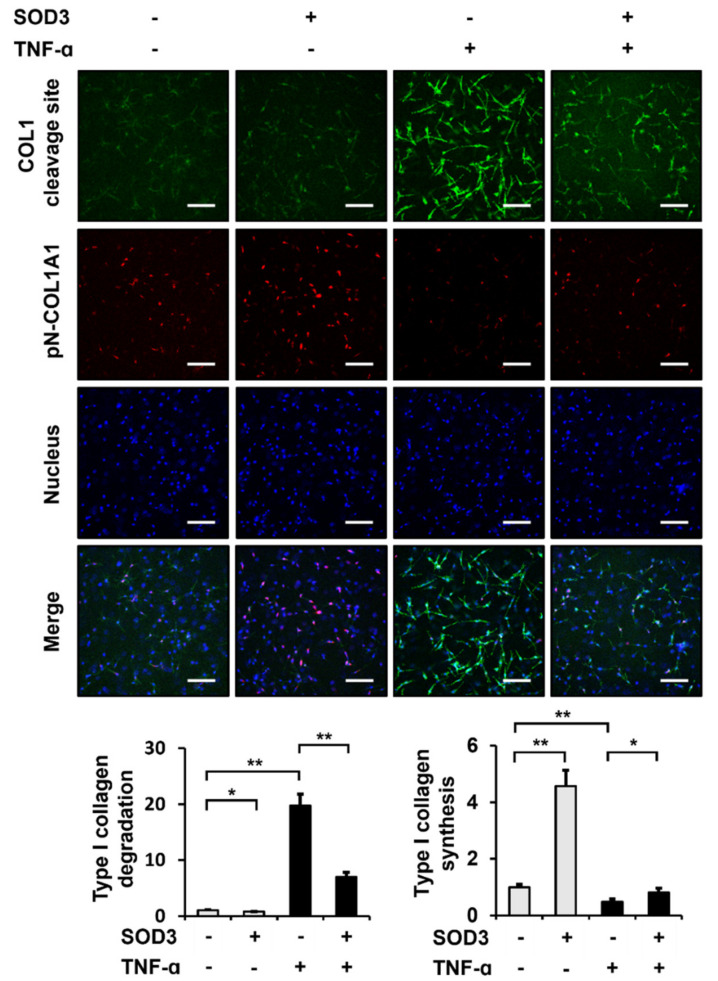
Effects of SOD3 on the degradation and synthesis of type I collagen in 3D cultures of fibroblasts. Foreskin fibroblasts embedded in a type I collagen matrix were incubated for 24 h with or without TNF-α (1 ng/mL) and SOD3 (3 µg/mL). The cells in the 3D culture were analyzed via immunofluorescence staining using rabbit anti-type I collagen cleavage-site and Alexa 488-conjugated anti-rabbit IgG antibodies for type I collagen degradation, mouse anti-pN-COL1A1 and Rhodamine Red X-conjugated anti-mouse IgG antibodies for type I collagen synthesis, and Hoechst 33258 for nuclear staining. The cells were analyzed via confocal microscopy. The fluorescence intensities were assessed using the ImageJ software and normalized to nuclear staining. Graphs show the fluorescence intensities for type I collagen degradation and synthesis relative to those in untreated cells. Each value represents the mean ± standard deviation of three independent experiments. * *p* < 0.05 and ** *p* < 0.01 vs. control. Magnification, ×100. Bar = 200 µm.

To analyze the effect of SOD3 on ECM integrity under in vivo-mimicking 3D conditions, we examined whether SOD3 affected the production and degradation of type I collagen in 3D cultures of foreskin fibroblasts. Immunofluorescence staining using type I collagen cleavage-site antibody showed that treatment with SOD3 significantly reduced the amount of cleaved 3/4 fragments of type I collagen to 77.1 ± 4.4% in the 3D culture of foreskin fibroblasts (Figure 6). TNF-α increased the amount of the type I collagen fragment to 1973.2 ± 205.9%. SOD3 still reduced the amount of the type I collagen fragment to 35.4 ± 4.1% in cells cultured in 3D gel in the presence of TNF-α. Furthermore, SOD3 significantly increased the amount of newly synthesized type I collagen to 456.4 ± 55.6% under 3D culture conditions (Figure 6). While TNF-α decreased the amount of nascent type I collagen to 48.1 ± 9.6% under the 3D culture conditions, SOD3 increased the amount of the type I collagen to 170.4 ± 31.2%. These data suggest that SOD3 reduces the breakdown of type I collagen and increases its synthesis of type I collagen. Taken together, we conclude that SOD3 plays an important role in maintaining ECM integrity under 3D culture conditions that mimic the dermis of the skin.

## 4. Discussion

ROS represent the number of reactive molecules and free radicals derived from molecular oxygen, including superoxide, hydrogen peroxide, hydroxyl radicals, hydroxyl ions, and nitric oxide [15,28,29,30]. The SOD family plays an essential physiological role in mitigating the deleterious effects of ROS [29]. In this study, to estimate the role of SOD family members in skin aging, we analyzed the mRNA levels of *Sod* members in the skin tissues of young and old mice. We found that the *Sod3* mRNA levels in the skin tissues of older mice were lower than those in young mice, but *SOD1* and *SOD2* mRNA levels were similar in the skin tissues of young and old mice. Interestingly, it was reported that plasma SOD3 levels were lower in adults than in children and youths, while blood SOD1 and SOD2 levels did not differ significantly between the groups of children, youths, and adults [31]. Although these results were for blood, they support the possibility that SOD3 expression, but not SOD1 and SOD2, is significantly altered with skin aging. Western blotting of mouse skin tissues and IHC staining of human skin tissues revealed that SOD3 protein levels decreased with age. Based on these results, we hypothesized that SOD3 most likely affects intrinsic skin aging.

SOD3, which is present in the extracellular space, reduces ROS levels in tissues [32,33]. It readily binds to negatively-charged ROS with high affinity through a positively-charged C-terminal heparin-binding domain [34]. Aging induces the accumulation of cellular damage, largely by the elevation of ROS in several types of cells, including mesenchymal stem cells. Ectopic expression of SOD3 rescues elderly mesenchymal stem cells by elevating ROS levels and cellular senescence [35]. In our study, we treated fibroblasts with 3 μg/mL recombinant SOD3, which showed the maximal effect on reducing MMP-1 secretion (Figure S2). Consistent with previous reports, SOD3 treatment decreased the intracellular ROS levels in fibroblasts. Since TNF-α, an inflammatory cytokine, is known to increase ROS levels [36,37] and 1 ng/mL TNF-α can induce MMP-1 expression in our experimental system [9], the antioxidant activity of SOD3 and its downstream effects in fibroblasts treated with and without 1 ng/mL TNF-α were analyzed.

An increase in ROS levels promotes the expression of MMPs, including MMP-1, and reduces the expression of type I collagen in many cell types, including dermal fibroblasts [38]. We observed that treatment with SOD3 decreased the secretion of MMP-1 and increased type I collagen secretion in fibroblasts. When cultured fibroblasts were treated with TNF-α, the secretion of MMP-1 was significantly increased compared to that in cells not treated with TNF-α, whereas the secretion of type I collagen was not significantly changed.

Using conventional and quantitative RT-PCR, we confirmed that SOD3 significantly decreased *MMP-1* mRNA levels and increased *COL1A1* and *COL1A2* mRNA levels. While TNF-α treatment significantly increased *MMP-1* mRNA levels, SOD3 significantly reduced the amount of *MMP-1* mRNA increased by TNF-α. The same result was observed for the promoter activities of *MMP-1*, *COL1A1*, and *COL1A2* as measured using the luciferase reporter assay. Therefore, we confirmed that SOD3-induced decrease in MMP-1 secretion and increase in type I collagen secretion were regulated at the transcriptional level.

We showed that SOD3 treatment suppressed *MMP-1* transactivation. It was known that the activation of NF-κB and AP-1 is important for the induction of MMP-1 [9] and that the inhibition or knockdown of signaling proteins that activate NF-κB and AP-1 downregulates MMP-1 expression [26]. ROS activates transcription factors, including AP-1 [39] and NF-κB [40,41]. The activation of NF-κB by ROS involves tyrosine phosphorylation of IκBα by IκB kinase and serine phosphorylation of NF-κB p65 [42,43,44,45]. The activation of p38 MAPK plays an important role in the transcriptional activation of NF-κB by acetylation of NF-κB p65 [46]. ROS also activates AP-1 through a series of signaling pathways [30,39,47]. ROS activate ERK, JNK, and p38 MAPK, which activate the transcription factor AP-1, composed of c-Jun and c-Fos, by phosphorylating c-Fos and c-Jun and recruiting them to target genes [26]. As expected from the ROS scavenging ability of SOD3, SOD3 increased IκB levels and reduced phosphorylation of NF-κB p65 and p38 MAPK, suggesting decreased activation of NF-κB and reduced phosphorylation of ERK, JNK, and p38 MAPK, which are important for the activation of AP-1.

We observed that the secretion of MMP-1 was upregulated following the treatment of fibroblasts with TNF-α. In addition, the treatment of fibroblasts with TNF-α reduced IκB levels and increased the phosphorylation of NF-κB p65, ERK1/2, JNK, and p38 MAPK. We demonstrated that SOD3 treatment reversed the phosphorylation of NF-κB p65, ERK1/2, JNK, and p38 MAPK, which were upregulated by TNF-α. These results suggest that SOD3 can suppress *MMP-1* transactivation and relieve inflammation by downregulating both the AP-1 and NF-κB signaling pathways that are activated under inflammatory conditions. Our finding is consistent with a report that SOD3 exerts anti-inflammatory effects by inhibiting the activation of TLR2, p38 MAPK, NF-κB, and NLRP3 inflammasome [48].

We also showed that SOD3 treatment transactivated the expression of type I collagen genes. NF-κB is known to suppress the expression of type I collagen by binding to NF-κB-responsive elements in the promoters of the *COL1A1* and *COL1A2* genes [49,50,51]. Since we observed that SOD3 reduced NF-κB activation, we hypothesized that the suppression of type I collagen expression by SOD3 could be due to NF-κB inhibition. We previously showed that 5 ng/mL TNF-α in fibroblasts reduced type I collagen secretion [21]. However, when fibroblasts were treated with 1 ng/mL TNF-α, NF-κB activation was observed, but an increase in type I collagen secretion and transactivation of type I collagen genes were not detected in fibroblasts. Recently, it was reported that SOD3 can promote type I collagen synthesis by activating AMPK and Nrf2/HO-1 cascades [52]. Therefore, we inferred that in the absence or weak inflammatory conditions, SOD3 could induce the expression of type I collagen through AMPK activation rather than NF-κB inhibition.

There is a notable decrease in dermal thickness and collagen strength in skin tissues with age and this process is associated with decreased collagen production and increased collagen degradation [53]. We found that the expression levels of SOD3 decreased with age. In addition, SOD3 decreased the expression levels of MMP-1 in cultured fibroblasts, while increasing the levels of type I collagen. When we analyzed fibroblasts cultured in 3D collagen gels mimicking the dermis, we found that SOD3 reduced MMP-1 secretion into the medium and significantly reduced the cleavage of type I collagen in a 3D matrix, which was observed using an anti-type I collagen cleavage site antibody [9], in the absence of TNF-α, and more evidently in the presence of TNF-α. SOD3 also significantly increased type I collagen biosynthesis in fibroblasts not treated with TNF-α, as observed using an anti-pN-COL1A1 antibody that detects nascent type I procollagen. In contrast to the results in 2D cultures, TNF-α reduced type I collagen biosynthesis in 3D cultures of fibroblasts, suggesting that TNF-α in 3D culture is more sensitive to fibroblasts than in 2D cultures. Nevertheless, SOD3 also increased type I collagen biosynthesis in TNF-α-treated fibroblasts cultured in a 3D matrix, suggesting that SOD3 improves ECM integrity by reducing collagen degradation and increasing collagen biosynthesis in a 3D matrix environment that mimics the dermis. In addition, since the expression of SOD3 decreases with age, we are certain that SOD3 plays a role in lowering intracellular ROS levels and maintaining ECM integrity in fibroblasts, thereby preventing and delaying skin aging. Given that SOD3 reduces collagen degradation and increases collagen production in fibroblasts under 3D culture conditions in the absence and presence of TNF-α, we propose that SOD3 has an effect of delaying both intrinsic and extrinsic skin aging.

## 5. Conclusions

Here, we found a decrease in SOD3 expression levels in mouse and human skin tissues with age. Treatment of fibroblasts with recombinant SOD3 decreased the intracellular ROS and MMP-1 expression levels, while increasing type I collagen expression levels. SOD3 reduced the activation of NF-κB and AP-1, which are required for MMP-1 induction. Under 3D culture conditions mimicking the dermis, SOD3 decreased the amount of type I collagen fragments produced by MMP-1, while increasing the amount of nascent type I procollagen. Based on these results, we propose that SOD3 prevents and retards skin aging by reducing the intracellular ROS levels and collagen breakdown, while promoting collagen production.

## Data Availability

Data is contained within the article and supplementary material.

## Supplementary Materials

The following are available online at https://www.mdpi.com/article/10.3390/antiox11050928/s1, Table S1: Primer sequences used for reverse transcription-polymerase chain reaction of mouse (m) *Sod* mRNAs and human (h) *COL1A1, COL1A2, MMP1*, and *GAPDH* mRNAs, Figure S1: Purification of recombinant human SOD3-His, Figure S2: Titration of the optimal SOD3 concentration to decrease MMP-1 secretion and increase type I collagen secretion in fibroblasts.
