# Supplementary material for: SOD3 Suppresses the Expression of MMP-1 and Increases the Integrity of Extracellular Matrix in Fibroblasts
Source: Antioxidants (Basel). 2022 May 9;11(5):928. doi: 10.3390/antiox11050928 (PMC9138143; doi:10.3390/antiox11050928)
Supplement: Supplementary file 1 [file antioxidants-11-00928-s001.zip › antioxidants-1695179-supplementary.pdf]

Supplementary Materials

SOD3 Suppresses the Expression of MMP-1 and Increases the Integrity of Extracellular Matrix in Fibroblasts

Jin Hyung Kim [1,†], Hae Dong Jeong [1,†], Min Ji Song [2], Dong Hun Lee [2,3,4], Jin Ho Chung [2,3,4,5] and Seung-Taek Lee [1,*]

[1] Department of Biochemistry, College of Life Science and Biotechnology, Yonsei University, Seoul 03722, Republic of Korea; qnt1313@yonsei.ac.kr (J.H.K.); ihaedong7@yonsei.ac.kr (H.D.J.)
[2] Department of Dermatology, Seoul National University College of Medicine, Seoul 03080, Republic of Korea; minjisong@snu.ac.kr (M.J.S.); ivymed27@snu.ac.kr (D.H.L.); jhchung@snu.ac.kr (J.H.C.)
[3] Laboratory of Cutaneous Aging Research, Biomedical Research Institute, Seoul National University Hospital, Seoul 03080, Republic of Korea
[4] Institute of Human-Environment Interface Biology, Seoul National University, Seoul 03080, Republic of Korea
[5] Institute on Aging, Seoul National University, Seoul 03080, Republic of Korea
* Correspondence: stlee@yonsei.ac.kr; Tel.: +82-2-2123-2703
† These authors contributed equally to this work.

Table S1. Primer sequences used for reverse transcription-polymerase chain reaction of mouse (m) *Sod* mRNAs and human (h) *COL1A1*, *COL1A2*, *MMP1*, *and GAPDH* mRNAs.

Gene Symbol	Nucleotide Sequence	Nucleotide Position	Annealing Temp. (°C)	GenBank #
m*SOD1*	5′- CAAGATGACTTGGGCAAAGGTGG -3′<br>5′- ACTGCGCAATCCCAATCACTCC -3′	466–488<br>559–538	55	NM_011434.2
m*SOD2*	5′- CCTCACTCACGGCCACATTG -3′<br>5′- ACAGCACCCCAGTCATAGTGC -3′	879–898<br>963–943	60	NM_013671.3
m*SOD2*	5′- CCAGGACACCTTAGTTAACCCAG -3′<br>5′- GGGGTTCTCTGAGAGCAGACTC -3′	1260–1282<br>1411–1390	65	NM_011435.3
h*COL1A1*	5′- ACAGCGTCACTGTCGATGGCTG -3′<br>5′- GGAGGGAGTTTACAGGAAGCAGACAG -3′	4341–4372<br>4522–4497	55	NM_000088
h*COL1A2*	5′- GAGGGCAACAGCAGGTTCACTTACAC -3′<br>5′-GTCAGCACCACCGATGTCCAA AG -3′	4044–4069<br>4196–4174	55	NM_000089
h*MMP1*	5′- GTACTGATATAATTTAGTTC -3′<br>5′- GTTATCCCTTGCCTATCTAG -3′	1656–1675<br>1908–1889	45	NM_002421
h*GAPDH*	5′- ACTGCTTAGCACCCCTGGCCA -3′<br>5′- TTGGCAGTGGGGACACGGAAG -3′	488–508<br>740–720	55	BC023632

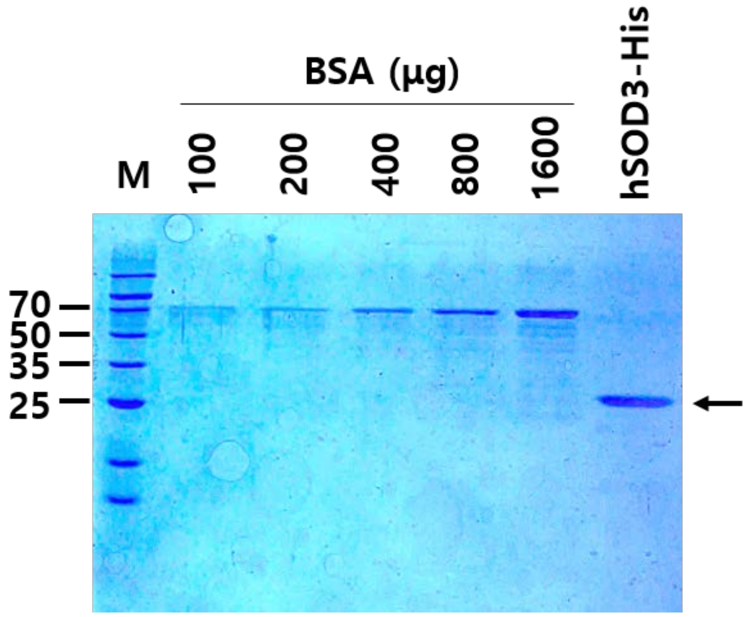

Figure S1. Purification of recombinant human SOD3-His.

Recombinant human (h) SOD3-His polypeptide was purified from the conditioned medium of HEK293 cells stably transfected with pcDNA3.1-hSOD3-His. The figure shows purified hSOD3-His polypeptide resolved by SDS-PAGE and stained with Coomassie brilliant blue. Various amounts of bovine serum albumin (BSA) were loaded to estimate the concentration of hSOD3-His polypeptide. Migration of molecular weight markers (M) is shown in the left lane of the gel.

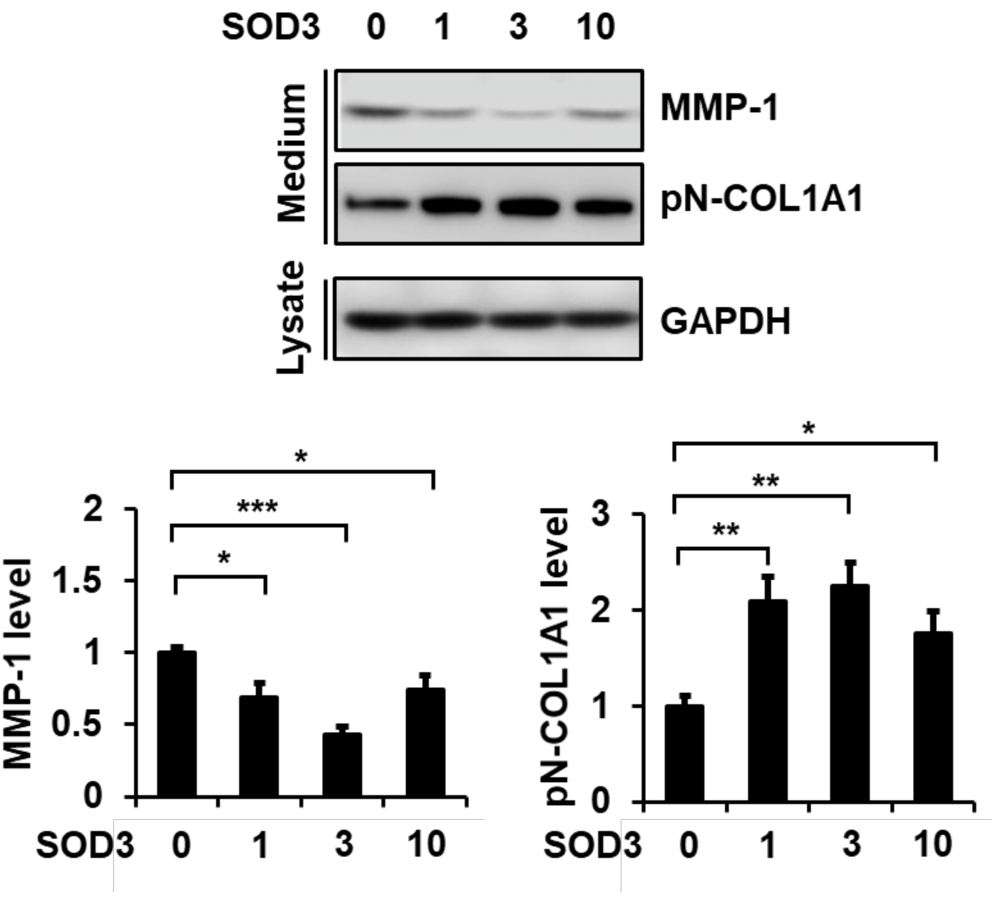

Figure S2. Titration of the optimal SOD3 concentration to decrease MMP-1 secretion and increase type I collagen secretion in fibroblasts.

Human foreskin fibroblasts were incubated in a serum-free medium in the presence of 0, 1, 3, and 10 μg/ml human SOD3-His for 24 h. The secreted levels of MMP-1 and type I collagen in conditioned media were assessed by western blot analysis using antibodies against MMP-1 and pN-COL1A1, respectively. The GAPDH level in cell lysates was monitored as a loading control. Graphs show levels of MMP-1 and type I collagen relative to the level in non-treated cells, which was quantified by image J software. Each value is the mean ± standard deviations of three independent experiments. *$p < 0.05$, **$p < 0.01$, and ***$p < 0.001$ vs. control.
